# Caregivers’ Knowledge and Experiences in Recognizing and Managing Dysphagia in Patients with Myopathy

**DOI:** 10.3390/clinpract15030061

**Published:** 2025-03-13

**Authors:** Maria Demetriou, Demetra Tziirkalli, Anastasios M. Georgiou

**Affiliations:** The “Cyprus Rehabilitating Aphasia and Dysphagia” (C-RAD) Lab, Department of Rehabilitation Sciences, School of Health Sciences, Cyprus University of Technology, 95 Irinis Str., 3041 Limassol, Cyprus

**Keywords:** swallowing, dysphagia, myopathy, professional caregivers, training

## Abstract

**Background:** Dysphagia is a common complication in myopathy, significantly impacting patients’ quality of life (QoL) and overall health. Caregivers play a critical role in identifying and addressing swallowing difficulties in this population. The main purpose of this study was to assess the knowledge and experiences of professional caregivers of patients with myopathy regarding the recognition and management of dysphagia in Cyprus. **Methods:** The study was designed as an anonymous, cross-sectional descriptive survey and involved 10 professional caregivers of patients with myopathy in Cyprus. **Results:** The most common dysphagia symptoms reported in myopathy patients were coughing, chewing difficulties, choking on fluids, and challenges with swallowing boluses. Only one caregiver reported difficulty managing swallowing issues, particularly in cases of reluctance to eat. Approximately 60% had received relevant training, primarily through workplace programs. Overall, caregivers did not perceive dysphagia as a significant burden. **Conclusions:** Dysphagia is a prevalent phenomenon in myopathy. The study reveals that caregivers of myopathy patients, regardless of their professional backgrounds, face hidden challenges in managing complex neurogenic dysphagia. They often misjudge the severity of the condition and overestimate their own competencies. Providing caregivers of patients with myopathy with targeted education would help them effectively manage swallowing difficulties associated with the condition. Encouragingly, our study also suggests that focused dysphagia education could reduce caregiver stress and enhance their overall well-being. Future efforts should concentrate on ensuring access to well-trained professionals, establishing specialized clinics, and promoting education to enhance MND-related dysphagia management and patient care.

## 1. Introduction

The term “myopathy” has Greek origins, derived from the words “myo-” meaning muscle (from the Greek “μῦς”) and “-pathy” meaning disease or condition (from the Greek “πάθος”). Among its most common symptoms are muscle weakness, muscle pain, and muscle stiffness. The complications of different types of myopathies are largely attributed to the progression of the disease, with dysphagia (also known as. swallowing disorders) being one of them [1].

Dysphagia is a relatively common problem in individuals with different NMDs [2], with its prevalence ranging between 30 and 84%, largely influenced by differences in the type and severity of each disorder [3]. Dysphagia in such patients is mainly identified in the oropharyngeal phase of swallowing which relies mostly on voluntary muscular activity of the structures of the oral cavity, pharynx, and upper oesophageal sphincter [2]. It may occur at the beginning [3] or at any other stage of the disease [4], with symptoms ranging from mild to severe, leading to complications such as malnutrition, dehydration, aspiration pneumonia, difficulty in managing oral medication, or even death [4,5]. In addition to serious complications, dysphagia can have social and psychological sequelae, significantly impacting the quality of life (QoL) of afflicted individuals [3,5]. Herein lies the crucial role of caregivers, who are responsible for assisting with the fundamental physical and psychological needs of affected patients, as well as supervising their daily activities [6]. The caregiver’s role can vary significantly, ranging from minimal involvement to extensive effort, and it may involve a short-term commitment or an indefinite period. In the context of NMDs, which are typically chronic conditions, caregiving often becomes a long-term commitment, necessitating adjustments in family dynamics, work obligations, and social activities [7].

In addition to informal caregivers, who are unpaid helpers such as family members, friends, and neighbors assisting adults with disabilities [8], the involvement of professional caregivers underscores the significance of specialized knowledge, training, and experience in providing essential care and support. Professional caregivers bring expertise in managing complex medical needs, ensuring consistent care delivery and enhancing the overall QoL for individuals with disabilities. Professional caregivers play a crucial role in improving patient outcomes and preventing secondary complications, while also complementing and strengthening the support offered by informal caregivers to form a holistic and effective caregiving network. Currently, there is a notable lack of data regarding the role of caregivers in managing dysphagia, particularly in the context of NMDs. A recent UK study highlights that caregivers of individuals with NMDs experience nearly three times the level of anxiety compared to patients with dysphagia, leading to increased vigilance and a heightened need to be present during mealtimes [3]. Additionally, caregivers express greater concern about symptom progression and weight loss, indicating they may recognize worsening symptoms at an earlier stage [3]. These findings underscore the need for targeted psychological support and proactive education to empower caregivers, helping them balance their responsibilities while improving patient outcomes. A Canadian study explored the experiences of caregivers of individuals with myotonic dystrophy (MD1) and dysphagia [9]. It was found that even though dysphagia can contribute to morbidity and mortality in individuals with DM1, caregivers did not view it as a primary concern. Instead, they focused on more debilitating symptoms, such as fatigue and weakness, and discussed the broader caregiving experience. This shift in caregiver priorities suggests the need for targeted interventions focused on these additional symptoms. Given the limited research on caregivers’ knowledge of dysphagia in patients with myopathy, it is essential to investigate their understanding of dysphagia within this population. In Cyprus, a small country with a population of under a million, no data currently exist on this topic. The primary aim of this study was to assess the knowledge and experiences of professional caregivers of patients with myopathy concerning the recognition and management of dysphagia in Cyprus.

The main research question that guided this study was as follows:

1. What problems do caregivers face in identifying and managing dysphagia in patients with myopathy?

The secondary research questions of this study were as follows:

1. To what extent are caregivers trained in recognizing and managing dysphagia in patients with myopathy?
2. To what extent does dysphagia affect/burden professional caregivers of patients with myopathy?

## 2. Materials and Methods

The study was approved by the Cyprus National Bioethics Committee with a reference number EEBK EΠ 2023.01.329, approval date: 14 December 2023. An anonymous, cross-sectional descriptive survey method was conducted to capture and analyze the knowledge and experiences of participants regarding the specific topic under investigation.

### 2.1. Instrument

The survey consisted of a self-report questionnaire, which was co-created by the research team, two members of which (M.D. and A.M.G.) have expertise in dysphagia. The survey instrument was developed based on a comprehensive review of the existing literature in neurogenic dysphagia. To ensure quality and reliability, the development process followed the CHERRIES checklist [10]. The questionnaire was developed electronically, using the Qualtrics platform (https://www.qualtrics.com/uk/, accessed on 11 November 2023) [11]. It covered two categories with a total of 21 questions. Most of the questions were multiple choice, apart from three questions that were displayed as a dropdown list and three matrix questions. Display and skip logic was used in some questions, ensuring that corresponding follow-up questions were shown based on the selected response. The first category focused on the sociodemographic characteristics of caregivers. It included questions about their gender, age, level of education, number of days and hours worked as professional caregivers for individuals with myopathy, and the total number of years they have been providing care. The second category was broader and included questions about feeding and swallowing. This category collected information on caregivers’ ability to observe, recognize, and report dysphagia symptoms, how they feed their patients, the viscosities in which individuals with myopathy experience difficulties, and the stability of their swallowing problems. In addition, caregivers were asked to report to what extent they face problems in managing the difficulties experienced by their patients and what techniques, if any, they use to compensate for them. Information was also collected on the extent of caregivers’ burden, the education/training they have received on identifying and/or managing feeding problems in patients with myopathy, and, lastly, which healthcare professionals they believe to be experts in dysphagia. The survey was conducted in Greek, as this is the official language of the Republic of Cyprus. The estimated time to complete the questionnaire was 10 min. In addition, the survey software allowed participants to complete the survey at their convenience time, enabling them to save their responses and return to them at any time before the survey’s deadline.

### 2.2. Pilot Phase

Prior to the distribution of the survey link and Qualtrics Quick Response (QR) code, a pilot phase was conducted involving a number of five caregivers to ensure the applicability of the survey and the ease of understanding the data. In particular, the participants were asked to read and complete the questionnaire, providing detailed feedback on the content and design of the tool developed in terms of the language used, the structure of it, etc. Once the researchers had reviewed the pilot participants’ recommendations one by one, appropriate modifications were made, and the final questionnaire was thus obtained. The pilot phase was carried out during a face-to-face training session for professional caregivers organized by the Cyprus Paraplegic Association (O.PA.C) once per year.

### 2.3. Participants

The study involved 10 professional caregivers of patients with myopathy in Cyprus.

### 2.4. Eligibility

Participants should be:

1. ≥18 years of age
2. Active professional caregivers of people with myopathy and dysphagia
3. Registered with the Care Service of the O.PA.C.
4. Providers of “Home Care Services” on behalf of the Cyprus Myopathy Association (MDA Cyprus).

### 2.5. Dissemination

The final version of the online survey was available for completion from February to March 2024. The link and QR code were distributed to potential participants via an O.PA.C officer, who was responsible for disseminating the survey to all registered caregivers of people with myopathy. The invitation to participate in the survey was made via email. This email was sent once approval had been obtained from the Cyprus National Bioethics Committee. A follow-up dissemination email was sent by the same person as a reminder two weeks after the survey was initially launched.

### 2.6. Statistical Analysis

Correlation analysis was not performed due to the small sample size of 10 participants, which lacked sufficient statistical power and increased the risk of Type I and Type II errors. Additionally, the sample consisted of only one man and nine women, which limits the generalizability of the findings and introduces potential gender bias. To ensure reliable and interpretable results within these constraints, only descriptive statistics were used, and these limitations should be considered when interpreting the findings. All statistical analyses were conducted using Microsoft Excel (Microsoft Corp., Redmond, WA, USA).

## 3. Results

### 3.1. Socio-Demographic Data

The survey was completed by 10 respondents, who reflected the total number of registered caregivers at O.PA.C and constituted the total study population. The ages of the participants ranged from 18 to 60. In terms of gender distribution, 90% of the study population were women and only 10% were men. Participants were mainly high school graduates (60%), with the remaining 40% being university alumni. All of the participants were employed as professional caregivers for people with myopathy, working between zero to four years, six days a week, for six hours each day. All demographic data can be found in Table 1.

### 3.2. Frequently Observed Dysphagia Signs

**Figure 1:**
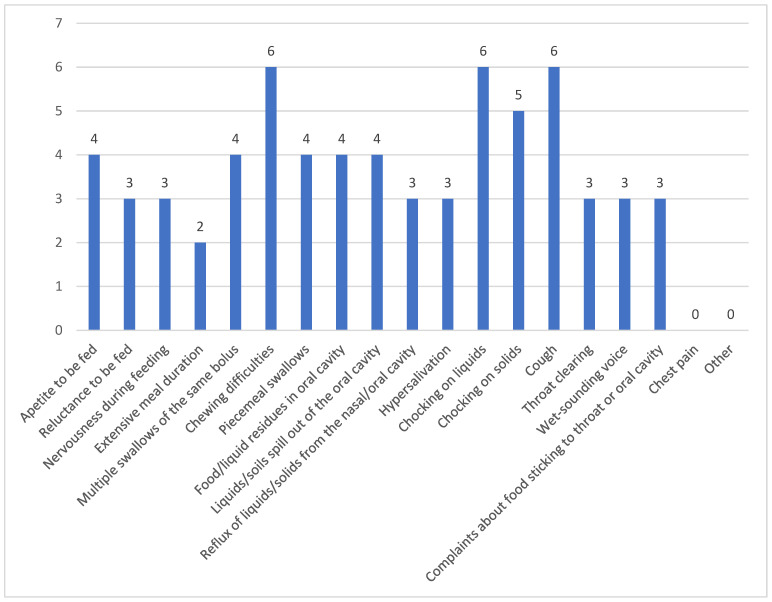
Dysphagia signs/symptoms observed by caregivers.

Study participants indicated coughing, chewing difficulties, and choking on liquids as the most commonly observed signs of dysphagia experienced by their patients during or after feeding, followed by choking on solid food (swallowing boluses). A detailed breakdown of all the indications reported by the caregivers can be found in Figure 1.

**Figure 2:**
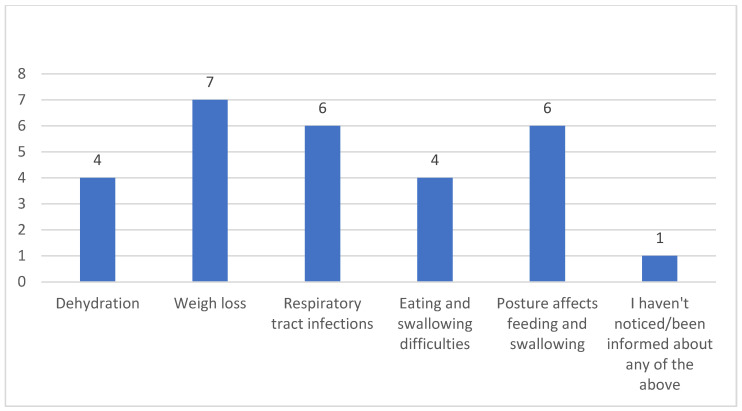
Caregivers’ awareness of dysphagia effects and associated challenges.

Caregivers were asked if they had observed or been informed about the effects of dysphagia, such as dehydration, weight loss, and respiratory infections. They were also questioned about difficulties related to feeding and swallowing at specific times of the day, as well as the importance of posture during swallowing. The majority of caregivers reported noticing or being informed about weight loss, respiratory infections, and the impact of posture on feeding and swallowing. However, one respondent indicated that they had not noticed or been informed about any of the listed issues (Figure 2).

### 3.3. Caregivers’ Patients Feeding Method

**Figure 3:**
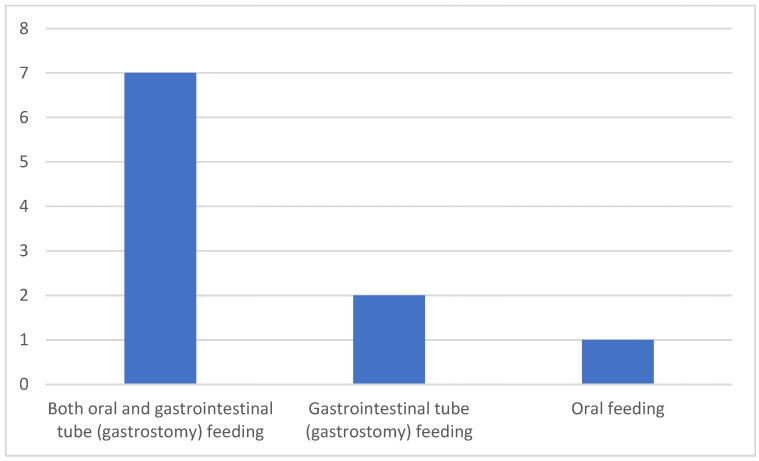
Feedings routes.

**Figure 4:**
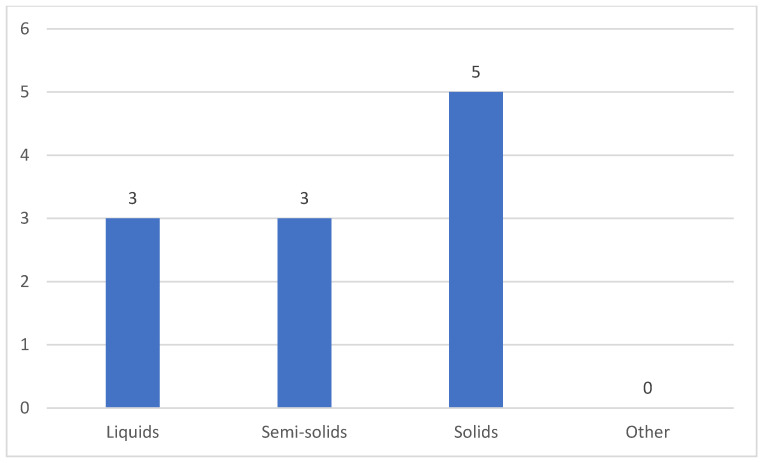
Food textures and fluid viscosities challenging for patients.

Seven caregivers provided care for patients who were fed both orally and via a gastrointestinal tube (gastrostomy), while two cared for patients exclusively fed through a gastrointestinal tube, and one cared for a patient who was solely orally fed (Figure 3). Among the eight caregivers of orally fed myopathy patients, five reported difficulties with swallowing specific food textures and fluid viscosities. Of these five, three observed challenges with liquids, solids, and semi-solids, whereas the remaining two noted difficulties only with solids (Figure 4).

### 3.4. Severity of Swallowing Difficulties and Their Management by Caregivers

**Figure 5:**
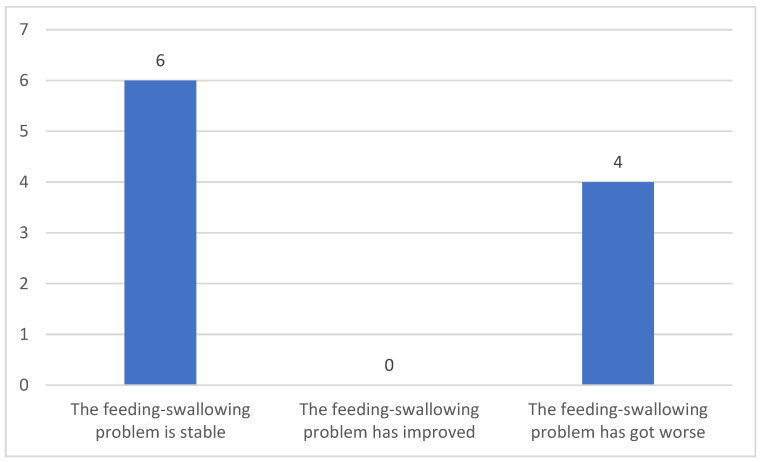
Status of the feeding–swallowing problem.

Sixty per cent of caregivers of people with myopathy reported that their patients’ swallowing difficulties were stable, while the remaining 40% stated that the problem had worsened (Figure 5). However, only one caregiver reported difficulty managing those difficulties. In particular, they noted that reluctance to eat is what they cannot manage (not shown in Figure 5).

### 3.5. Management Strategies Used by Caregivers

**Figure 6:**
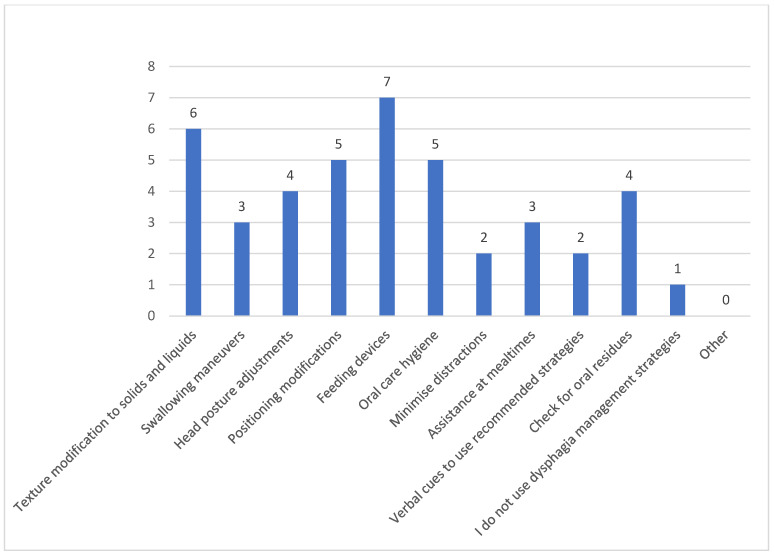
Dysphagia management strategies used by caregivers.

Feeding devices (e.g., feeding syringes, adaptive utensils, sippy cups, lip cups, straw cups, feeding cups with lids), texture modifications to food and liquids, positioning modifications (e.g., patient sitting at a 90-degree angle), as well as oral hygiene/care were the most prevalent strategies used by caregivers to manage their patients’ swallowing difficulties (Figure 6).

### 3.6. Burden of Caregivers

**Figure 7:**
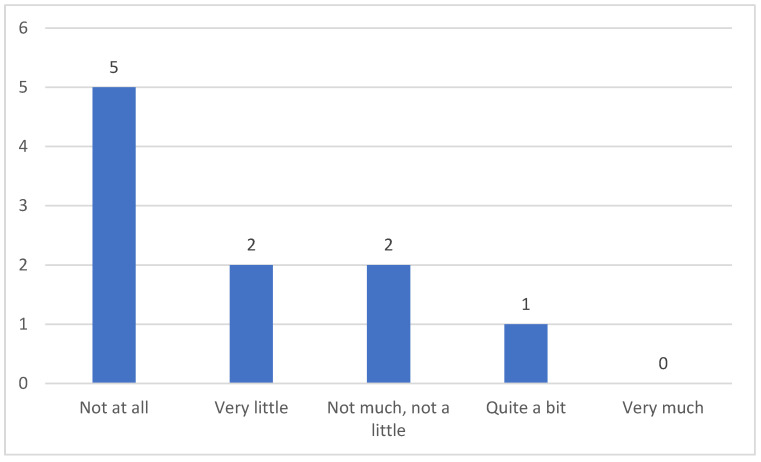
Caregivers’ burden.

Half of the participants said that their role as caregivers was not burdened by their patients’ dysphagia. Two caregivers reported being neither greatly not slightly affected, two more said they were affected very little, while only one participant was affected quite a bit (Figure 7).

### 3.7. Education/Training of Caregivers

**Figure 8:**
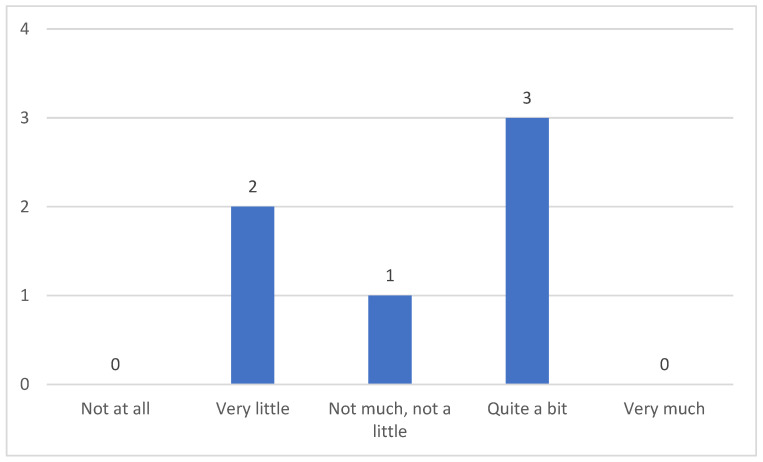
Level of satisfaction of caregivers regarding the training/education they received.

Of the ten participants, six had received some kind of education/training on the recognition and/or management of swallowing problems in patients with myopathy. Their training/education was provided through seminars and workplace training programs. Of the individuals who received training/education, three reported they were significantly satisfied, two participants were slightly satisfied, while one participant reported they were neither very nor slightly satisfied (Figure 8).

**Figure 9:**
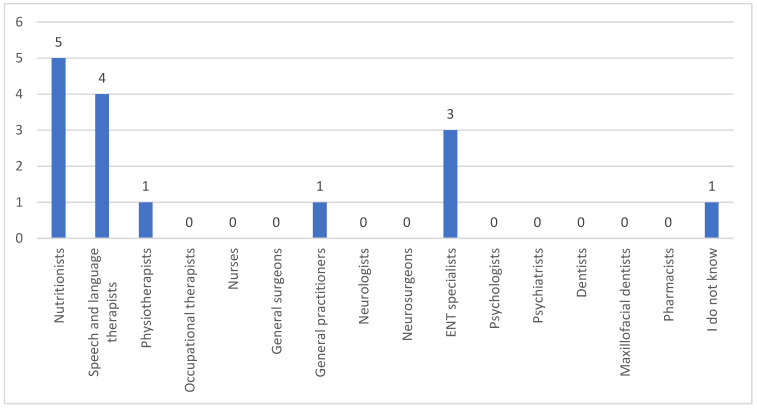
Healthcare professionals considered dysphagia experts by caregivers.

Following a question about which healthcare professionals the participants considered experts in dysphagia, nutritionists emerged as the predominant answer, with speech and language therapists (SLTs) and otolaryngologists following (Figure 9). Physiotherapists and general practitioners were also mentioned. One participant reported that they did not know which professionals were specialists in dysphagia.

**Figure 10:**
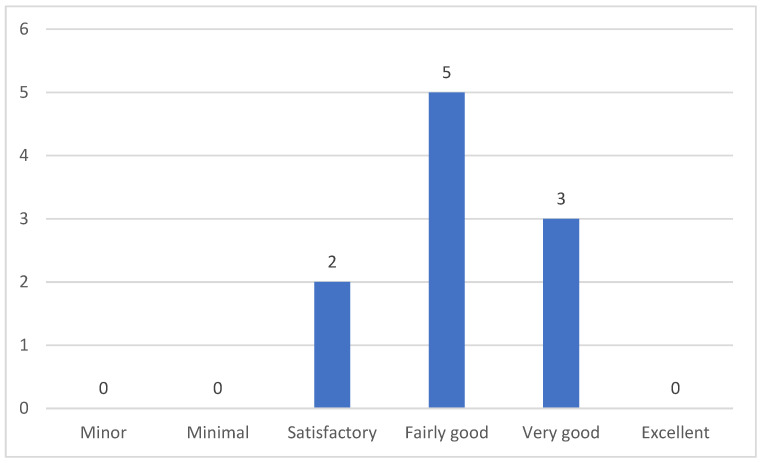
Caregivers’ assessment of their knowledge about feeding/swallowing of patients with myopathies.

In general, caregivers rated their knowledge about feeding and swallowing of patients with myopathy as fairly good (50%), very good (30%), and satisfactory (20%) (Figure 10).

## 4. Discussion

This study was set to investigate the knowledge and experiences of professional caregivers of patients with myopathy regarding the recognition and management of dysphagia in Cyprus. Due to the limited research on caregivers’ knowledge of dysphagia in myopathy patients, exploring their understanding of this condition is essential. This study is therefore highly relevant for improving care for individuals with myopathy and offering better support to their caregivers.

The primary research question guiding this study focused on the challenges faced by caregivers in identifying and managing dysphagia symptoms in patients with myopathy. Regarding dysphagia identification, the fact that nine out of ten caregivers were able to recognize its symptoms or were informed about them indicates a relatively high level of awareness among professional caregivers of NMD-related dysphagia. In particular, nine participants were able to identify coughing, chewing difficulties, choking from drinking fluids, and difficulty swallowing boluses as the most frequently observed signs of dysphagia. These findings are consistent with several studies that highlight similar symptoms as common indicators of dysphagia (e.g., [3,12,13,14]). Additionally, the ability of six caregivers to identify stability in the dysphagia symptoms and four who recognized worsening symptoms reflects a degree of sensitivity to changes in the patient’s condition. However, the differing levels of recognition also suggest variability in caregivers’ understanding of the progression of dysphagia in myopathy patients, which may impact timely intervention and care. Previous research [3] showed that just over half of caregivers of people with NMDs were unaware of the possibility of dysphagia as a consequence of this disorder, whereas those who had knowledge of dysphagia in relation to NMDs were mainly self-educated. With regard to dysphagia management, the finding that nine caregivers felt confident in managing myopathy-related dysphagia, while one caregiver identified difficulty in managing reluctance to eat, underscores the challenges caregivers encounter. The reluctance to eat is a critical component of dysphagia management that may not only affect nutritional intake but also patient well-being, highlighting an area where caregivers may need additional support or training. Overall, it is believed that, although caregivers were capable of identifying symptoms of dysphagia and recognizing its worsening, their confidence in managing the condition reflects an underestimation of dysphagia’s complexity. Neurogenic dysphagia is highly complex, often involving silent aspiration, which complicates detection and management. Without adequate clinical bedside evaluation and frequent instrumental assessments, it is questionable whether caregivers can accurately identify and manage dysphagia. For example, myositis-related dysphagia shows a wide incidence range from 10% to 73%, with aspiration pneumonia being the leading cause of death [15]. This raises a crucial question: How can caregivers assess the physiology and biomechanics of the pharyngeal phase without specialized training or tools? This is not feasible. Therefore, it is important that caregivers receive adequate training not only to recognize dysphagia but also to understand when and to whom they should refer patients.

The above findings highlight the second research focus of our study: examining the extent to which caregivers are trained in recognizing and managing dysphagia in myopathy. The findings revealed that 60% of participants reported receiving relevant training, primarily through workplace training programs and seminars. This is expected, as these participants are professional caregivers. Interestingly, among those who received training, three participants expressed being fairly satisfied, two were slightly satisfied, and one reported neither being very nor slightly satisfied. To summarize, while nine caregivers felt confident in managing dysphagia, only three considered their skills to be fairly satisfactory. Additionally, four caregivers have never received training in dysphagia management. This raises the question: How can those four, without any training, feel confident in managing dysphagia? What is crystal clear is that confidence in managing dysphagia without proper training is problematic, as it reflects both an underestimation of the condition’s complexity and an overestimation of one’s own skills. As mentioned earlier, dysphagia is a complex condition that requires specialized expertise for accurate detection and management. Several studies emphasize the need for caregivers to receive training tailored to managing feeding and swallowing difficulties in patients with myopathy, as the level of training often varies depending on the caregivers’ work environment [3,16,17,18]. A collaborative approach involving patients, caregivers, and healthcare professionals is considered the most effective strategy [19]. Without advanced training, caregivers may face challenges in identifying critical issues such as silent penetration or aspiration, which are crucial for preventing severe complications. Therefore, assessing the effectiveness of existing training programs and identifying gaps in caregivers’ knowledge or practical skills is essential. This evaluation can help determine whether additional training is necessary to enhance care for patients with myopathy-related dysphagia.

Importantly, and as it relates to our third research question, the overall results indicated that caregivers of patients with myopathy do not appear to be significantly burdened by their patients’ dysphagia. Notably, half of the study participants reported that their caregiving role was not impacted by their patients’ swallowing difficulties, while only one participant indicated being significantly affected. This finding contrasts with previous studies suggesting caregivers bear a heavy burden [20,21], highlighting the complexity and variability in experiences. A recent systematic review found that caregivers of patients with myopathy experienced decreased health-related QoL (HRQOL), reported poor quality of sleep, impaired family functioning, depression, anxiety, and other symptoms [22]. Those findings are in line with Landfeldt et al. [23], showing that caring for a person with myopathy can entail significant burden and reduced HRQOL. It is likely that the differences in perceived caregiver burden between our study and previous research can be attributed to the involvement of informal caregivers and the focus on specific types of myopathy in earlier studies. In comparison, our findings suggest that dysphagia training could play a key role in reducing caregiver stress and burden, ultimately enhancing the caregiver experience.

Last but not least, the respondents emphasized that nutritionists possess the most expertise in dysphagia. While they are indeed important for addressing nutritional needs, decisions regarding feeding methods and swallowing difficulties are coordinated by SLTs, at least in Cyprus, whose roles are globally recognized. These specialists lead multidisciplinary teams to ensure safe, appropriate, and effective dysphagia assessment and management.

This study was primarily exploratory, offering initial insights into the experiences and knowledge of caregivers of individuals with myopathy-related dysphagia. However, several limitations should be acknowledged. A significant limitation is the low response rate, mainly attributed to the convenience sampling method, relying on the Cyprus Paraplegic Association (O.PA.C.), the only organization of its kind in Cyprus. Furthermore, the sample was predominantly female, with only one male participant, which limits the generalizability of the findings and introduces potential gender bias. While the study provides valuable insights into the experiences of professional caregivers, its findings cannot be broadly generalized due to these limitations. Future studies should address this limitation by using more diverse sampling methods to determine the number of professional caregivers involved in the care of patients with NMDs and how many of them manage dysphagia, thus enhancing the generalizability and robustness of the findings. Furthermore, the lack of detailed patient characteristics, such as the type of myopathy, severity of dysphagia, and gastrostomy status, limits the depth of analysis. Additionally, the absence of specific tools used to evaluate dysphagia severity restricts a thorough understanding of how these factors influence caregivers’ knowledge and experiences. Future research should address these gaps by incorporating detailed patient data and standardized assessment tools to further explore the relationship between dysphagia severity and caregivers’ experiences and knowledge. Furthermore, should the survey be repeated in the future, it could be beneficial to focus on a specific type of myopathy rather than on patients diagnosed with myopathy in general. This approach would allow for a more detailed exploration of the particular challenges and characteristics associated with that specific subtype. Importantly, given the likelihood that the sample size in Cyprus may not increase in the future, this underscores the need for broader research efforts. Collaboration with international studies would help confirm and expand upon these initial findings, providing a more comprehensive understanding of the experiences and knowledge of caregivers managing myopathy-related dysphagia.

Despite the small sample size, this study plays a crucial role in advancing the care of patients with myopathy-related dysphagia by identifying key gaps in caregiver education. Disseminating these findings both locally and internationally will enhance the scientific health community’s understanding of caregiver challenges, highlighting the need for a more collaborative approach to dysphagia management. Ultimately, this can lead to improved quality of care and quality of life for both patients and their caregivers.

## 5. Conclusions

Dysphagia is a prevalent issue in NMDs, leading to significant challenges and complications for patients. Our findings indicate that caregivers of myopathy patients, irrespective of their professional backgrounds, encounter unrecognized challenges in managing complex neurogenic dysphagia. They frequently underestimate the condition’s complexity while overestimating their own knowledge and skills. Adequate education tailored to caregivers is essential for effective dysphagia management in myopathy. On a positive note, our study suggests that targeted dysphagia training can play a crucial role in reducing caregiver stress and burden, ultimately improving their experience. Moving forward, it is essential to ensure access to well-trained professionals with the knowledge and skills necessary to address the often-overlooked needs of caregivers of NMD patients. Establishing specialized clinics and multidisciplinary teams focused on dysphagia, along with implementing targeted education and training programs, presents a valuable opportunity to raise awareness and improve management strategies in NMDs. Such initiatives have the potential to enhance dysphagia management and treatment outcomes, leading to more effective and improved patient care.

## Figures and Tables

**Table 1:** Demographic characteristics of professional caregivers for individuals with myopathy.

Gender	Age Range	Education Level	Working Hours per Day	Working Days per Week	Years of Caregivers’ Experience
Female	46–50	High School	6	6	0–1
Female	46–50	High School	6	6	2–3
Female	31–35	High School	6	6	1–2
Female	41–45	University	6	6	0–1
Female	56–60	High School	6	6	1–2
Female	56–60	University	6	6	1–2
Female	36–40	High School	6	6	0–1
Female	18–25	University	6	6	0–1
Male	46–50	University	6	6	1–2
Female	41–45	High School	6	6	2–3

## Data Availability

The data sets utilized during this study are available from the corresponding author upon reasonable request.
